# Parasites of domestic and wild animals in South Africa. XLIX. Ticks (Acari: Ixodidae) infesting white and black rhinoceroses in southern Africa

**DOI:** 10.4102/ojvr.v84i1.1301

**Published:** 2017-01-30

**Authors:** Ivan G. Horak, Christiaan R. Boshoff, David V. Cooper, Christoper M. Foggin, Danny Govender, Alan Harrison, Guy Hausler, Markus Hofmeyr, J. Werner Kilian, Duncan N. MacFadyen, Pierre J. Nel, Dean Peinke, David Squarre, David Zimmermann

**Affiliations:** 1 Department of Veterinary Tropical Diseases, University of Pretoria, South Africa; 2 Wild Game, Gravelotte, South Africa; 3 EKZN Wildlife, St Lucia, South Africa; 4 Victoria Falls Wildlife Trust, Victoria Falls, Zimbabwe; 5 Scientific Services, SANParks, Skukuza, South Africa; 6 Institute of Biological and Environmental Sciences, University of Aberdeen, United Kingdom; 7 Veterinary Wildlife Services, SANParks, Skukuza, South Africa; 8 Okuakuejo Rest Camp, Etosha National Park, Namibia; 9 Department of Research and Conservation, E Oppenheimer & Son, South Africa; 10 Department of Economic Development, Tourism and Environmental Affairs, Bloemfontein, South Africa; 11 Eastern Cape Parks & Tourism Agency, East London, South Africa; 12 Wildlife Veterinary Unit, Zambia Wildlife Authority, Zambia; 13 Veterinary Wildlife Services, SANParks, Port Elizabeth, South Africa

## Abstract

The objectives of the study were to determine the species composition of ticks infesting white and black rhinoceroses in southern Africa as well as the conservation status of those tick species that prefer rhinos as hosts. Ticks were collected opportunistically from rhinos that had been immobilised for management purposes, and 447 white rhinoceroses (*Ceratotherium simum*) and 164 black rhinoceroses (*Diceros bicornis*) were sampled in South Africa, 61 black rhinos in Namibia, 18 white and 12 black rhinos in Zimbabwe, and 24 black rhinos in Zambia. Nineteen tick species were recovered, of which two species, *Amblyomma rhinocerotis* and *Dermacentor rhinocerinus*, prefer rhinos as hosts. *A. rhinocerotis* was collected only in the north-eastern KwaZulu-Natal reserves of South Africa and is endangered, while *D. rhinocerinus* is present in these reserves as well as in the Kruger National Park and surrounding conservancies. Eight of the tick species collected from the rhinos are ornate, and seven species are regularly collected from cattle. The species present on rhinos in the eastern, moister reserves of South Africa were amongst others *Amblyomma hebraeum*, *A. rhinocerotis*, *D. rhinocerinus*, *Rhipicephalus maculatus*, *Rhipicephalus simus* and *Rhipicephalus zumpti*, while those on rhinos in the Karoo and the drier western regions, including Namibia, were the drought-tolerant species, *Hyalomma glabrum*, *Hyalomma rufipes*, *Hyalomma truncatum* and *Rhipicephalus gertrudae*. The species composition of ticks on rhinoceroses in Zambia differed markedly from those of the other southern African countries in that *Amblyomma sparsum*, *Amblyomma tholloni* and *Amblyomma variegatum* accounted for the majority of infestations.

## Introduction

Historically white rhinoceroses (*Ceratotherium simum*) were present from South Africa in the south to the coastal regions of north-eastern Africa in the north (Skinner & Chimimba 2005). However, with the arrival of European settlers in southern Africa, as well as hunters, naturalists and travellers, the numbers of white rhinos in this region rapidly dwindled. Towards the end of the 19th century the last white rhinos were shot in Zimbabwe and Botswana, while in South Africa their numbers had declined to approximately 20–50 animals in north-eastern KwaZulu-Natal. The proclamation of the Hluhluwe and Imfolozi Game Reserves in 1895 and the Mkuze Game Reserve in 1912 in KwaZulu-Natal is considered to have rescued them from extinction in South Africa (Skinner & Chimimba 2005). Their numbers have steadily increased since then and in 1961 the Natal Parks Board initiated a project on the relocation of rhinoceroses from the reserves under its control to the Kruger National Park and other state and provincially controlled reserves, as well as to privately owned reserves lying within the rhinos’ former distribution range. Although the number of white rhinos in South Africa now exceeds 18 000, the ever-increasing rate at which they are being poached may soon surpass their birth rate.

The numbers of black rhinoceroses (*Diceros bicornis*) declined as rapidly as those of white rhinoceroses. From a situation in which they were present on the slopes of Table Mountain in 1652, to where the proclamation of the Hluhluwe, Imfolozi and Mkuze game reserves in KwaZulu-Natal rescued them from extinction in this country (Skinner & Chimimba 2005). Numbers on the continent are believed to have declined from approximately 100 000 in the early 1960s to only 2410 in 1995. Since then careful management and relocations have seen their numbers on the continent increase to 4880 by the end of 2010 (Cumming, Du Toit & Stuart 1990; Knight, Balfour & Emslie 2013), with most present in the north-eastern KwaZulu-Natal reserves, the Kruger National Park and the Etosha National Park.

Both rhinoceros species are infested with a greater array of ornate ixodid ticks than any other mammal species in East and southern Africa. Most of these are brightly coloured members of the genus *Amblyomma*, and include *Amblyomma eburneum*, *Amblyomma gemma*, *Amblyomma hebraeum*, *Amblyomma personatum*, *Amblyomma rhinocerotis*, *Amblyomma sparsum*, *Amblyomma tholloni* and *Amblyomma variegatum*. Rhinos also harbour *Dermacentor rhinocerinus*, an ornate tick that is for all practical purposes a specific parasite of these animals. They are also infested by three of the four *Rhipicephalus* species that are ornate, *Rhipicephalus humeralis*, *Rhipicephalus maculatus* and *Rhipicephalus pulchellus*.

A number of tick surveys conducted in Africa have included rhinoceroses amongst various other animal species sampled. Collections made from black rhinoceroses in Tanzania yielded 18 tick species, including six species in the genus *Amblyomma* as well as *D. rhinocerinus*, and two ornate *Rhipicephalus* spp. (Yeoman & Walker 1967). Twenty-one tick species were identified in collections from black rhinoceroses in Kenya; these included seven *Amblyomma* spp., *D. rhinocerinus* and three ornate *Rhipicephalus* spp. (Walker 1974). Ten tick species were present in collections made from white and black rhinoceroses in Zimbabwe, and three *Amblyomma* spp. and *D. rhinocerinus* were recovered (Norval 1983; Norval & Colborne 1985). In a checklist of ticks that infest large mammals in the KwaZulu-Natal reserves in South Africa, 11 species were reported on rhinoceroses, these included three *Amblyomma* spp. (one of them doubtful), *D. rhinocerinus* and *R. maculatus* (Baker & Keep 1970). Collections from two white and four black rhinoceroses sampled at various localities in South Africa yielded nine tick species, including *A. hebraeum*, *D. rhinocerinus* and *R. maculatus* (Knapp et al. 1997).

It is generally not realised that should rhinoceroses go extinct, a multitude of smaller creatures will also disappear. Amongst these are the rhinoceros-specific ticks, *A. personatum*, *A. rhinocerotis*, *Cosmiomma hippopotamensis* and *D. rhinocerinus*, the flies *Rhinomusca dutoiti*, *Rhinomusca brucei* and *Gyrostigma rhinocerontis*, as well as a horde of rhino-specific nematodes and an even greater number of commensal protozoal species. The extinction of rhinos will thus destroy a whole ecosystem of parasites and commensals.

With the exception of the study by Knapp et al. (1997), no surveys devoted solely to rhinoceroses and the ticks that infest them have been conducted in southern Africa. The objective of the present investigation is to address this shortcoming. As a consequence, a number of surveys aimed at determining the species composition of ticks that infest these animals in the sub-continent were initiated.

## Methods

Participants in the study collected ticks opportunistically from rhinoceroses immobilised for management purposes. Particular attention was paid to the ears, the axilla, inner thighs, peri-anal region and the tail brush. Although this collection procedure represented the ideal, any ticks collected from rhinos were included in the study. More detailed or complete collections were not possible because of the time these would take, causing additional anaesthetic stress on the animals. Ticks collected from each rhino were placed in separate bottles or plastic vials containing 70% ethyl alcohol or undiluted methylated spirits. A label written in pencil, indicating the rhino’s species, its name or identity (if known), gender, the date, the locality at which it was immobilised and the identity of the person responsible for collecting ticks, was placed with the ticks in the bottle or vial. This was not always possible, and several labels were attached to the outside of the containers. The ticks were identified and counted using a stereoscopic microscope.

The identities and total numbers of adult ticks collected from white and black rhinoceroses across southern Africa are summarised in tabular format. They are also tabulated according to the regions in which they were collected from rhinos within South Africa. A separate table has been created for ticks collected in Namibia, Zimbabwe and Tanzania. The regional distributions of the 19 tick species collected from rhinoceroses in southern Africa are represented in a mosaic format.

The conservation status of the ticks that prefer rhinos as hosts and the introduction of ticks into non-endemic habitats or reintroduction into endemic habitats are discussed, as is the role of rhinos as maintenance hosts of ticks that are vectors of diseases of domestic livestock or wildlife species.

### Ethical considerations

No rhinos were immobilised for the sole purpose of collecting ticks. Collection was incidental to other management procedures necessitating immobilisation.

## Results

### South Africa

The tick species recovered from a total of 465 white and 261 black rhinoceroses across southern Africa, and the number of animals infested with each species are summarised in Table 1. Twelve tick species, including the nymphs of *Rhipicephalus appendiculatus*, were collected from white rhinoceroses, with *A. hebraeum* present in the majority of collections, followed by *D. rhinocerinus*, *Hyalomma truncatum* and *Hyalomma rufipes*. Sixteen species were recovered from black rhinoceroses, and *A. hebraeum* was the most commonly collected tick, followed by *H. rufipes* and *H. truncatum*. The two rhino species harboured ten tick species in common.

**TABLE 1:** Ixodid ticks collected from white and black rhinoceroses in southern Africa.

Ticks collected from rhinoceroses	Tick species	Number infested	% infested	Number of ticks recovered		
				Males	Females	Total
**South Africa and Zimbabwe**						
White rhinoceroses (*n* = 465)	*Amblyomma hebraeum*	382	82.2	1797	1474	3271
	*Amblyomma rhinocerotis*	8	1.7	11	7	18
	*Amblyomma tholloni*	3	0.6	1	2	3
	*Dermacentor rhinocerinus*	153	32.9	478	112	590
	*Hyalomma rufipes*	71	15.3	218	59	277
	*Hyalomma truncatum*	86	18.5	244	89	333
	*Rhipicephalus evertsi evertsi*	3	0.6	2	3	5
	*Rhipicephalus follis*	5	1.1	19	8	27
	*Rhipicephalus gertrudae*	7	1.5	3	12	15
	*Rhipicephalus maculatus*	17	3.7	234	116	350
	*Rhipicephalus simus*	27	5.8	50	56	106
**South Africa, Namibia, Zimbabwe and Zambia**						
Black rhinoceroses (*n* = 261)	*Amblyomma gemma*	1	0.4	3	2	5
	*Amblyomma hebraeum*	141	54.0	847	528	1375
	*Amblyomma rhinocerotis*	3	1.1	7	5	12
	*Amblyomma sparsum*	23	8.8	88	60	148
	*Amblyomma tholloni*	15	5.7	18	15	33
	*Amblyomma variegatum*	14	5.4	58	23	81
	*Dermacentor rhinocerinus*	10	3.8	36	10	46
	*Hyalomma glabrum*	4	1.5	8	1	9
	*Hyalomma rufipes*	96	36.8	312	151	463
	*Hyalomma truncatum*	75	28.7	516	266	782
	*Rhipicephalus follis*	19	7.3	67	47	114
	*Rhipicephalus gertrudae*	9	3.4	38	14	52
	*Rhipicephalus longiceps*	1	0.4	0	1	1
	*Rhipicephalus maculatus*	10	3.8	121	54	175
	*Rhipicephalus neumanni*	2	0.8	0	2	2
	*Rhipicephalus simus*	44	16.9	161	67	228
	*Rhipicephalus zumpti*	2	0.8	3	0	3

**FIGURE 1:**
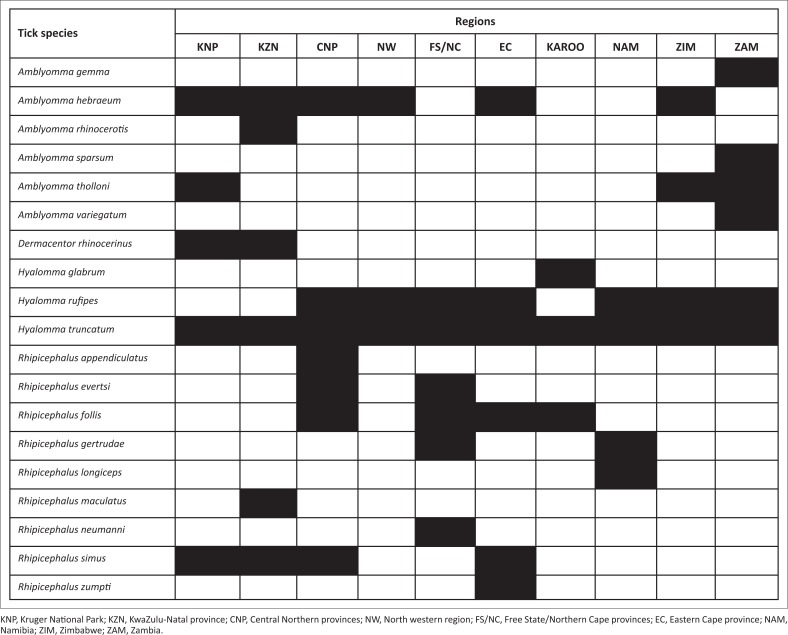
The regional distribution of ticks that infest rhinoceroses in southern Africa.

The species and numbers of ticks collected from 303 white rhinoceroses in the Kruger National Park and surrounding conservancies and from nine black rhinoceroses in the park, as well as those collected from 49 white rhinos and 20 black rhinos in the north-eastern KwaZulu-Natal reserves, are summarised in Table 2 and the regions in which the ticks were collected are represented in Figure 1. Five species were collected from white rhinos and four from black rhinos in the Kruger National Park. *Amblyomma hebraeum* was present in the majority of collections made from either rhino species, followed by *D. rhinocerinus*. Three white rhinos were infested with *A. tholloni*, a tick whose adults prefer elephants as hosts.

**TABLE 2:** Ixodid ticks collected from rhinoceroses in the Kruger National Park and surrounding conservancies, and in the north-eastern KwaZulu-Natal reserves.

Ticks collected from rhinoceroses	Tick and host species	Number infested	% infested	Number of ticks recovered		
				Males	Females	Total
**Kruger National Park and surrounding conservancies**						
White rhinoceroses (*n* = 303)	*Amblyomma hebraeum*	294	97.0	1180	957	2137
	*Amblyomma tholloni*	3	1.0	1	2	3
	*Dermacentor rhinocerinus*	145	47.0	428	96	524
	*Hyalomma truncatum*	31	10.0	41	15	56
	*Rhipicepalus simus*	20	7.0	27	17	44
Black rhinoceroses (*n* = 9)	*Amblyomma hebraeum*	9	100.0	33	24	57
	*Dermacentor rhinocerinus*	6	66.7	10	3	13
	*Hyalomma truncatum*	1	0.3	1	0	1
	*Rhipicepalus simus*	1	11.1	3	0	3
**North-eastern KwaZulu-Natal reserves**						
White rhinoceroses (*n* = 49)	*Amblyomma hebraeum*	49	100.0	273	267	540
	*Amblyomma rhinocerotis*	8	16.3	11	7	18
	*Dermacentor rhinocerinus*	8	16.3	50	16	66
	*Rhipicephalus maculatus*	17	34.7	234	116	350
	*Rhipicepalus simus*	5	10.2	3	3	6
Black rhinoceroses (*n* = 20)	*Amblyomma hebraeum*	19	95.0	108	97	205
	*Amblyomma rhinocerotis*	3	15.0	7	5	12
	*Dermacentor rhinocerinus*	4	20.0	26	7	33
	*Hyalomma truncatum*	1	5.0	1	0	1
	*Rhipicephalus maculatus*	10	50.0	121	54	175
	*Rhipicepalus simus*	2	10.0	2	1	3

Five tick species were recovered from the white rhinos in the KwaZulu-Natal reserves and six from the black rhinos. *Amblyomma hebraeum* was present in the majority of collections followed by *R. maculatus* on both white and black rhinos, while some animals of both species were infested with the rhinoceros specific ticks *A. rhinocerotis* and *D. rhinocerinus* (Figure 1).

The tick species collected from rhinoceroses in the Free State, Northern Cape province, North West province, western Limpopo province, and the central and south-eastern region of the Limpopo province and north-eastern Gauteng are summarised in Table 3. *Hyalomma rufipes* and *H. truncatum* were the most frequently collected ticks from both rhino species in the Free State and Northern Cape. The collections of *A. hebraeum* from white rhinoceroses in the Free State were made from animals that had recently been introduced into the province from regions in which the tick was present and do not represent established populations. Most white rhinos in eastern North West province and western Limpopo province were infested with *A. hebraeum,* and it was the only species collected from black rhinoceroses.

**TABLE 3:** Ixodid ticks collected from rhinoceroses in the Free State, Northern Cape province, north-western parks and central region of two northern provinces of South Africa.

Ticks collected from rhinoceroses	Tick and host species	Number infested	% infested	Number of ticks recovered		
				Males	Females	Total
**Central region of the northern provinces**						
White rhinoceroses (*n* = 11)	*Amblyomma hebraeum*	8	72.7	183	193	376
	*Hyalomma rufipes*	6	54.5	18	2	20
	*Hyalomma truncatum*	1	9.1	1	0	1
	*Rhipicephalus eversti evertsi*	2	18.2	2	2	4
	*Rhipicephalus follis*	4	36.4	6	5	11
	*Rhipicephalus simus*	2	18.2	20	36	56
**North-western parks of South Africa**						
White rhinoceroses (*n* = 6)	*Amblyomma hebraeum*	5	83.3	24	13	37
	*Hyalomma rufipes*	2	33.3	3	1	4
	*Hyalomma truncatum*	2	33.3	2	0	2
Black rhinoceroses (*n* = 12)	*Amblyomma hebraeum*	12	100.0	50	19	69
**Free State and Northern Cape provinces**						
White rhinoceroses (*n* = 78)	*Amblyomma hebraeum*	8	10.3	15	11	26
	*Hyalomma rufipes*	62	79.5	194	56	250
	*Hyalomma truncatum*	52	66.7	200	74	274
	*Rhipicephalus evertsi evertsi*	1	1.3	0	1	1
	*Rhipicephalus follis*	1	1.3	13	3	16
	*Rhipicephalus gertrudae*	7	9.0	3	12	15
Black rhinoceroses (*n* = 27)	*Hyalomma rufipes*	25	92.6	82	30	112
	*Hyalomma truncatum*	24	88.9	120	61	181
	*Rhipicephalus follis*	3	11.1	4	1	5
	*Rhipicephalus gertrudae*	3	11.1	3	2	5
	*Rhipicephalus neumanni*	2	7.4	0	2	2

Ticks were also collected from a dead white rhinoceros originating from a reserve in the southern central region of Limpopo province and presented for necropsy at the Faculty of Veterinary Science, Pretoria University, Onderstepoort. Because it was dead, considerably more ticks were collected from it than from other white rhinos that were temporarily immobilised. Ticks were also collected from eight white rhinos in the south-eastern region of Limpopo province and two animals in north-eastern Gauteng. We consider all these animals to have come from the central region of two of the northern provinces of South Africa and the numbers and species of ticks collected from them are summarised in Table 3. In addition to *A. hebraeum* and *H. rufipes*, they harboured *Rhipicephalus evertsi evertsi* and *Rhipicephalus follis* and the dead animal was infested with a large number of *Rhipicephalus simus*. Three of the eleven animals were infested with seven nymphs of *R. appendiculatus*.

The species and numbers of ticks collected from rhinoceroses in the Great Fish River Nature Reserve and the Addo Elephant National Park in the Eastern Cape province, and the Mountain Zebra and the Karoo National Parks are summarised in Table 4. All but one of the black rhinoceroses examined in the Eastern Cape province were infested with *A. hebraeum*, and a substantial number of collections of *R. follis* and particularly *R. simus* were also made. The rather rare species, *Rhipicephalus zumpti,* was also present. Four of the black rhinoceroses examined in the Mountain Zebra and Karoo National Parks were infested with *Hyalomma glabrum* and five with *H. truncatum* and *R. follis*.

**TABLE 4:** Ixodid ticks collected from black rhinoceroses in the Great Fish River Nature Reserve and the Addo Elephant National Park, Eastern Cape province, and the Mountain Zebra and Karoo National Parks.

Ticks collected from rhinoceroses	Tick and host species	Number infested	% infested	Number of ticks recovered		
				Males	Females	Total
**Eastern Cape province**						
Black rhinoceroses (*n* = 90)	*Amblyomma hebraeum*	89	98.9	558	344	902
	*Hyalomma rufipes*	5	5.6	4	2	6
	*Hyalomma truncatum*	5	5.6	11	11	22
	*Rhipicephalus follis*	11	12.2	30	26	56
	*Rhipicephalus simus*	41	45.6	156	66	222
	*Rhipicephalus zumpti*	2	2.2	3	0	3
**Mountain Zebra and Karoo National Parks**						
Black rhinoceroses (*n* = 6)	*Hyalomma glabrum*	4	66.7	8	1	9
	*Hyalomma truncatum*	5	83.3	17	3	20
	*Rhipicephalus follis*	5	83.3	33	20	53

### Namibia

The tick species collected from black rhinoceroses in three regions of Namibia are summarised in Table 5. The rhinos in the Etosha National Park were infested with *H. rufipes* and *H. truncatum*, the Damaraland rhinos with *H. rufipes* and one animal with the very rare *Rhipicephalus longiceps*, while the rhinos in the Hardap Nature Reserve harboured *H. rufipes*, *H. truncatum* and *Rhipicephalus gertrudae*, an assemblage of ticks fairly similar to that on white and black rhinoceroses in the Free State and Northern Cape provinces (Figure 1).

**TABLE 5:** Ixodid ticks collected from rhinoceroses in Namibia, Zimbabwe and Zambia.

Ticks collected from rhinoceroses	Tick species and hosts	Number infested	% infested	Number of ticks recovered		
				Males	Females	Total
**Three localities in Namibia**						
Black rhinoceroses: Etosha (*n* = 33)	*Hyalomma rufipes*	30	91.0	102	59	161
	*Hyalomma truncatum*	32	97.0	350	183	533
Black rhinoceroses: Damaraland (*n* = 22)	*Hyalomma rufipes*	22	100.0	103	53	156
	*Rhipicephallus longiceps*	1	4.5	1	0	1
Black rhinoceroses: Hardap (*n* = 6)	*Hyalomma rufipes*	5	83.3	7	5	12
	*Hyalomma truncatum*	5	83.3	14	7	21
	*Rhipicephalus gertrudae*	6	100.0	35	12	47
**Zimbabwe**						
White rhinoceroses (*n* = 18)	*Amblyomma hebraeum*	18	100.0	122	33	155
	*Hyalomma rufipes*	1	5.6	3	0	3
Black rhinoceroses (*n* = 12)	*Amblyomma hebraeum*	12	100.0	98	44	142
	*Amblyomma tholloni*	1	8.3	0	1	1
	*Hyalomma rufipes*	3	25.0	6	2	8
	*Hyalomma truncatum*	1	8.3	1	0	1
**Zambia**						
Black rhinoceroses (*n* = 24)	*Amblyomma gemma*	1	4.2	3	2	5
	*Amblyomma sparsum*	23	95.8	88	60	148
	*Amblyomma tholloni*	14	58.3	18	14	32
	*Amblyomma variegatum*	14	58.3	58	23	81
	*Hyalomma rufipes*	6	25.0	8	0	8
	*Hyalomma truncatum*	1	4.2	1	1	2

### Zimbabwe

The results of collections from rhinos in the Malilangwe reserve in south-eastern Zimbabwe are summarised in Table 5. All the animals were infested with *A. hebraeum*. A single collection of *A. tholloni* was made from a black rhinoceros.

### Zambia

The tick species present on black rhinoceroses examined in the North Luangwa National Park in Northern province differed substantially from those of the other southern African animals (Table 5). *Amblyomma sparsum* was the dominant species with all but one animal being infested. A number of collections of *A. tholloni* and *A. variegatum* were also made (Figure 1). The Zambian rhinos had originally come from the Kruger National Park, Marakele National Park and the KwaZulu-Natal and Eastern Cape parks.

## Discussion

### Amblyomma gemma

The only rhinos infested with *A. gemma* were those examined in Zambia (Figure 1). Neither Theiler (1962) nor Walker and Olwage (1987) include Zambia within the distribution range of *A. gemma*. In neighbouring Tanzania, its distribution is almost entirely limited to semi-arid bush and thicket and by rainfall between 38 mm and 76 mm, with a few records close to the north-eastern border of Zambia (Yeoman & Walker 1967). Its collection now from a black rhinoceros in Northern province, Zambia is possibly a new locality record.

The preferred hosts of the adults of *A. gemma* are large domestic and wild herbivores (Theiler 1962; Walker 1974; Yeoman & Walker 1967). Yeoman and Walker (1967) record collections from four of eight rhinoceroses examined in Tanzania, and Walker (1974) reports collections from 28 of 54 black rhinoceroses sampled in Kenya. Considering that only one of the 24 rhinoceroses examined in Zambia was infested, it may imply that the distribution of *A. gemma* in northern Zambia is tenuous, or that collections were made during the wrong season. Nymphs have been recovered from helmeted guineafowls and Cape hares (Yeoman & Walker 1967).

### Amblyomma hebraeum

*Amblyomma hebraeum* is present along the southern and eastern seaboard of South Africa, from approximately Port Elizabeth in the west to southern Mozambique in the east. It also occurs in the northern provinces of South Africa, south-eastern Botswana, and southern and north-western Zimbabwe (Walker & Olwage 1987). The collections from rhinos in the north-eastern, eastern and south-eastern regions of South Africa and south-east Zimbabwe (Figure 1) all lie within its known geographical distribution range (Norval 1983; Spickett 2013; Walker & Olwage 1987). It has been introduced into the Grassland biome of Free State province on white rhinoceroses but is unlikely to survive there (Horak et al. 2015). However, should it be introduced into the Savanna biome in the north of the same province its establishment there is a distinct possibility.

The adults of this brightly coloured tick infest cattle, sheep and goats as well as the larger wildlife species, while its immature stages infest the same hosts as the adults, but also hares, the larger ground frequenting birds and tortoises (Dower, Petney & Horak 1988; Horak, Golezardy & Uys 2007; Horak et al. 1987). In Zimbabwe, Norval (1983) recorded *A. hebraeum* in 15 of 19 tick collections made from white rhinoceroses, and in the present survey the 30 rhinoceroses examined in that country were all infested. The large proportion of rhinos that were infested in most of the regions in which *A. hebraeum* occurs, as well as the large numbers of adult ticks that have been collected from rhinos when total collections were made (Knapp et al. 1997), is a clear indication that rhinos must be considered as one of the preferred hosts of this species.

*Amblyomma hebraeum* is the most effective vector of *Ehrlichia ruminatium*, the causative organism of heartwater in cattle, sheep and goats and some wildlife species (Norval & Horak 2004). It is also the vector of *Rickettsia africae*, the causative organism of African tick bite fever in humans (Kelly 2001).

### Amblyomma rhinocerotis

The first two ticks with a South African origin to be described were *Amblyomma rhinocerotis* and *Amblyomma sylvaticum*. *Amblyomma rhinocerotis* had been collected from a rhinoceros at the Cape of Good Hope and *A. sylvaticum* from angulate tortoises, and both were described by De Geer in 1778. By all accounts, the adults of *A. rhinocerotis* are host-specific parasites of white and black rhinoceroses, and were probably present on these animals in the coastal and wooded inland regions from Cape Town in the south-west to the Kruger National Park in the north-east of South Africa. However, by the turn of the 19th century *A. rhinocerotis* and its hosts possibly only survived in the north-eastern region of KwaZulu-Natal, South Africa. Subsequent to the review on the ticks infesting larger wildlife in the KwaZulu-Natal reserves by Baker and Keep in 1970, there have been no reports, until now, of its presence in South Africa. In Zimbabwe, Duncan (1989) visually estimated that there were between 100 and 500 or more adult *A. rhinocerotis* on 16 of 18 black rhinoceroses prior to their treatment with an acaricide and relocation from the Zambezi Valley to safer habitats in the centre of the country. There have been no subsequent reports of its occurrence in that country. In their review of the ixodid ticks which they believe to be endangered, Mihalca, Gherman and Cozma (2011) regard *A. rhinocerotis* as ‘critically endangered’ if black rhinoceroses were its only hosts within a particular region, whereas on white rhinoceroses it is ‘near threatened’.

Despite numerous translocations of rhinos from the north-eastern KwaZulu-Natal reserves, *A. rhinocerotis* has failed to become established elsewhere, perhaps because rhinos are treated with an acaricide before translocation. Its precarious survival even in the KwaZulu-Natal reserves is highlighted by the fact that only 11 of the 69 rhinoceroses examined there were infested and that only 18 male and 12 female ticks were collected. Not one of the 303 white or 9 black rhinoceroses examined in the Kruger National Park was infested, nor any of the 96 black rhinoceroses in the Eastern Cape and Karoo (Figure 1). Some of the latter animals were examined in the Albany Thicket Biome, a habitat possibly suitable for the survival of the tick. Nor were any of the rhinos examined in Namibia, Zimbabwe or Zambia infested.

### Amblyomma sparsum

Walker and Olwage (1987) have plotted the overall distribution of *A. sparsum* with most records coming from Tanzania and Kenya, some from northern and north-western Zimbabwe and a single record from south-eastern Zambia. According to Theiler and Salisbury (1959), there is an isolated record of *A. sparsum* from Grootfontein, Namibia. Walker (1991) elaborates on this record, stating that it was a male tick collected by a Government Veterinary Officer in 1933. No subsequent collections have been made, and we now believe *A. sparsum* to be extinct in Namibia.

The adults of *A. sparsum* infest two remarkably different groups of hosts. The one group comprises tortoises, monitor lizards and the larger species of snakes and the other rhinoceroses and buffaloes (Walker 1974; Yeoman & Walker 1967). Norval (1983) recorded a total of 619 male and 175 female *A. sparsum* in collections from 66 black rhinoceroses in north-western Zimbabwe, and Duncan (1989) visually estimated there to be between 10 and 50 ticks on 16 of 18 rhinoceroses he examined in the same region. The collection now of *A. sparsum* from 23 of 24 black rhinoceroses in North Luangwa National Park, Zambia indicates that it is well established there (Figure 1).

Norval and MacKenzie (1981) successfully transmitted *E. ruminantium* to sheep by means of *A. sparsum* nymphs that had fed as larvae on an infected sheep. However, transmission via adult ticks that had fed either as larvae or as nymphs on infected sheep, failed.

### Amblyomma tholloni

According to Walker and Olwage (1987), the distribution of *A. tholloni* is linked to that of its preferred host, the African elephant, *Loxodonta africana* in southern, East, and Central Africa, as well as in some of the southern countries in West Africa. Sixteen collections of *A. tholloni* were made from elephants in the Kruger National Park during the present survey, while in Zimbabwe all 29 elephants and 22 of 24 hippopotamuses examined for ticks, were infested (Norval 1983). The recovery of *A. tholloni* from three white rhinoceroses in the Kruger National Park, a single black rhinoceros in Zimbabwe and 14 black rhinoceroses in Zambia, implies that it may use rhinos as alternative hosts to elephants and hippopotamuses. Mihalca et al. (2011) list *A. tholloni* as coendangered with its elephant hosts as ‘vulnerable’ to extinction.

MacKenzie and Norval (1980) experimentally transmitted *E. ruminantium* to sheep by means of *A. tholloni* nymphs that had been fed as larvae on an infected sheep, and by adult ticks that had been fed as nymphs on infected sheep. Because of their preference for elephants, the adults of *A. tholloni* are unlikely to play a role in the transmission of *E. ruminantium* in the field. In addition, elephants and domestic livestock very rarely share the same habitat, thus free-living *A. tholloni* questing for hosts are unlikely to be present in the absence of elephants. However, MacKenzie and Norval (1980) reported that cattle, sheep and goats at the Rekomitji Research Station situated in a game reserve in the Zambezi Valley of Zimbabwe were frequently infested with *A. tholloni* larvae and nymphs and that cases of heartwater were recorded in domestic livestock at the research station in the absence of any of the known vectors.

### Amblyomma variegatum

The geographical distribution of *A. variegatum* includes north-western Zimbabwe, the Zambezi Strip in north-eastern Namibia, much of Zambia and thence northwards into sub-Saharan Africa (Walker & Olwage 1987). Adult ticks prefer cattle as well as large wild herbivores as hosts, while the immature stages infest the same hosts as the adults, and also hares and the larger species of ground-feeding birds (Petney, Horak & Rechav 1987; Theiler 1962). Theiler (1962) reports adult ticks on white and black rhinoceroses, while Walker (1974) records collections from five of 54 black rhinoceroses in Kenya. The large proportion of black rhinoceroses infested with *A. variegatum* in Zambia suggests that these animals are amongst the preferred hosts for adult ticks. *Amblyomma variegatum* is an effective vector of *E. ruminantium* (Norval & Horak 2004) and also of *R. africae* (Kelly 2001).

### Cosmiomma hippopotamensis

Joseph Burke, a British naturalist made the first ever collection of *C*. *hippopotamensis* from a rhinoceros or a hippopotamus about 20 km to the north-west of Pretoria in 1840, and a male tick and female tick from this collection were described by Denny in 1843. No collections have been made in South Africa since then (Apanaskevich et al. 2013). In Namibia, Bezuidenhout and Schneider (1972) made the last recorded collections of *C. hippopotamensis* in Kaokoland in the north-west of the country in 1971. They collected 114 adult ticks from vegetation along footpaths used by rhinoceroses to get to water and successfully fed some of these ticks on a rhinoceros calf. One of the three female ticks that engorged on the calf laid a large batch of fertile eggs from which larvae hatched and these were used in studies to determine the life cycle of the tick (Apanaskevich et al. 2013). The translocation of rhinos from Kaokoland to the Etosha National Park and settlement of humans in the north-west of the country has probably contributed to the disappearance of *C. hippopotamensis* in Namibia. We think it is now extinct in both South Africa and Namibia, and perhaps in Africa. In their review of ixodid tick species coendangered with their hosts, Mihalca et al. (2011) consider *C. hippopotamensis*’ status as ‘vulnerable’.

### Dermacentor rhinocerinus

Only two of the 35 *Dermacentor* species that occur world-wide are present in the Afrotropical region, *Dermacentor circumguttatus* and *D. rhinocerinus* (Guglielmone et al. 2014). The adults of *D. circumguttatus* prefer elephants as hosts and those of *D. rhinocerinus* prefer white and black rhinoceroses (Guglielmone et al. 2014; Knapp et al. 1997). *Dermacentor rhinocerinus* was first described by Denny in 1843 from a male specimen collected from a black rhinoceros in South Africa (Keirans 1993). *Dermacentor rhinocerinus* is widespread in Africa, but as rhino populations decrease or disappear because of poaching, its distribution range is diminishing (Keirans 1993). Mihalca et al. (2011) consider *D. rhinocerinus* to be ‘critically endangered’ should black rhinos be its only hosts, and ‘near threatened’ on white rhinos.

With the possible exception of pockets in north-eastern KwaZulu-Natal, *D. rhinocerinus*, with its rhino hosts, had probably become extinct in the rest of South Africa, including the region now known as the Kruger National Park, by the end of the 19th and beginning of the 20th centuries. We believe that *D. rhinocerinus* has been re-introduced into the Kruger National Park with rhinoceroses from the KwaZulu-Natal parks (Braack et al. 1995). Its prevalence on rhinos in the park and surrounding conservancies now appears to be greater than that in the KwaZulu-Natal nature reserves. The larvae and nymphs of *D. rhinocerinus* feed on rodents (Horak & Cohen 2001), for which they probably quest from the soil surface or from the base of tufts of grass. The brightly coloured questing adult ticks are commonly encountered high up on thick grass stems along the verges of roads and game paths, wherever there are dense populations of rhinos in the Kruger National Park.

Since 1933, when it was collected from a black rhinoceros at Grootfontein, no collections of *D. rhinocerinus* have been made in Namibia (Walker 1991). We now consider it to be extinct there. During the 1970’s and 80’s *D. rhinocerinus* was present in the south-east and south-west of Zimbabwe and at several localities in the Zambezi Valley in the north-west of the country (Duncan 1989; Norval & Colborne 1985). This may no longer be true after the poaching and consequent precautionary translocation of rhinoceroses that has taken place. None of the 30 rhinoceroses examined in south-eastern Zimbabwe in the present study, were infested. Although *D. rhinocerinus* was not present amongst the ticks collected from rhinos in Zambia, it has previously been reported as occurring there (Keirans 1993).

### Hyalomma glabrum

*Hyalomma glabrum*, previously referred to as *Hyalomma marginatum turanicum*, was reinstated as a valid species by Apanaskevich and Horak (2006). It is the most colourful of the three *Hyalomma* species that occur in South Africa. In addition to the ivory coloured band that encircles the distal margin of each segment of its legs, the dorsal surface of each segment is coated with a strip of ivory coloured enamelling. This is especially distinct on the segments of the hind legs. *Hyalomma glabrum* is a strictly South African tick and is the only *Hyalomma* sp. whose distribution is confined to the southern hemisphere. It is present in Nama Karoo and Succulent Karoo Biomes in the Eastern, Western and Northern Cape provinces (Apanaskevich & Horak 2006). It would seem that *H. glabrum* and *H. rufipes* are mutually exclusive in their habitat preferences.

The adults of *H. glabrum* have a preference for large herbivores and large numbers have been collected from Cape mountain zebras and especially eland in the Mountain Zebra National Park. Its immature stages have been collected from scrub hares and birds in the park (Horak et al. 1991a). The presence of adult ticks on black rhinoceroses after their re-introduction into the Mountain Zebra National Park and the Karoo National Park more than a century after their disappearance in the Karoo is thus not unexpected. The collections from black rhinoceroses are first records on these animals.

### Hyalomma rufipes

*Hyalomma rufipes* is a drought-tolerant species and with the exception of the north-eastern regions of Mpumalanga and Limpopo provinces, the eastern regions of KwaZulu-Natal, the eastern Free State and the southern regions of the Western Cape province, it is present throughout South Africa (Spickett 2013). In agreement with this pattern of distribution, no *H. rufipes* were collected from rhinoceroses in the Kruger National Park and surrounding conservancies, nor in the north-eastern parks of KwaZulu-Natal. *Hyalomma rufipes* was replaced by *H. glabrum* in the Karoo and their occurrence seems to be mutually exclusive (Figure 1). *Hyalomma rufipes* was the predominant species in the central and western Free State and Northern Cape province, and was present on black rhinos in all three regions in which collections were made in Namibia.

The preferred hosts of the adults of *H. rufipes* are large domestic and wild ruminants, particularly cattle, giraffes and eland (Dreyer, Fourie & Kok 1998; Horak et al. 2007), while the immature stages infest Cape hares, scrub hares and birds (Horak & Fourie 1991; Van Niekerk, Fourie & Horak 2006). It was the second most prevalent species collected from rhinos in southern Africa in the present study (Figure 1), and rhinos must be considered as one of the preferred hosts of adult *H. rufipes*.

*Hyalomma rufipes* is a vector of *Babesia occultans*, the causative organism of benign babesiosis in cattle, with infection passing transovarially from one generation of adults to the next (Gray & De Vos 1981). It is also the most effective vector in South Africa of the virus causing Crimean-Congo haemorrhagic fever in humans (Swanepoel et al. 1983).

### Hyalomma truncatum

*Hyalomma trunctum* is a drought-tolerant species and is absent in the moist eastern as well as the colder Highveld regions of South Africa (Spickett 2013). It is the only species that was collected in every region in which rhinos were examined (Figure 1). A large number of collections were made from rhinos in the Free State and Northern Cape provinces, it was the only *Hyalomma* species collected from rhinos in the Kruger National Park and the predominant species on animals in the Etosha National Park.

The adults of *H. truncatum* prefer large herbivores as hosts and large numbers have been collected from giraffes and eland (Horak et al. 2007), while the immature stages infest Cape hares, scrub hares and murid rodents (Horak & Fourie 1991; Horak et al. 1991a). Judging by the large number of rhinos that were infested and that ticks were present on them in every region included in the present study, rhinos must be considered as one of the preferred hosts of the adults of *H. truncatum*.

*Hyalomma truncatum* is a vector of *Babesia caballi*, the cause of equine piroplasmosis, with infection passing transovarially from one generation of adults to the next. The females also produce an epitheliotrophic toxin responsible for sweating sickness in calves (Norval & Horak 2004).

### *Rhipicephalus* species

The ticks, *R. follis*, *R. gertrudae* and *R. simus*, are similar in appearance but the denseness of punctations on the conscuta of the males and scuta of the females varies (Walker, Keirans & Horak 2000). Although there is some overlap, their geographical distributions differ (Walker et al. 2000). *Rhipicephalus follis* is moderately punctate and is associated with mountainous terrain mainly east of longitude 24º. *Rhipicephalus gertrudae* is heavily punctate and is present in drier regions west of this longitude and in the winter rainfall regions of the Western and Northern Cape provinces where summers are hot and dry. It is also present in Namibia. But for four irregular rows of large punctations, the conscutum of *R. simus* males is smooth. It is widespread in the northern, eastern and south-eastern regions of South Africa. The collections made from rhinoceroses in the various regions reflect the distributions of these three ticks. All of them prefer monogastric animals such as zebras, warthogs and now also rhinoceroses, as well as the larger carnivores as hosts, but buffaloes, elands and cattle may also be infested (Horak et al. 2007). Their larvae and nymphs prefer murid rodents as hosts (Walker et al. 2000).

A single collection of *R. longiceps* was made from a black rhinoceros in Namibia. It is the only *Rhipicephalus* species, which, relative to its size, has long mouthparts (Walker et al. 2000). Its distribution is confined to Namibia and Angola and in total very few collections have been made (Walker et al. 2000). These collections include ticks from warthogs and a giraffe and now also a rhinoceros (Horak et al. 1992; Horak et al. 1983).

*Rhipicephalus maculatus* is the only ornate *Rhipicephalus* species present in southern Africa. It is present in a broad strip of coastal mosaic vegetation and adjacent woodlands from Durban in KwaZulu-Natal northwards to Somalia (Walker et al. 2000). It has short but sturdy mouthparts and its preferred hosts are large mammals with thick hides, such as elephants, rhinoceroses, warthogs, bushpigs and buffaloes (Baker & Keep 1970; Horak, Boomker & Flamand 1991b; Horak et al. 2007; Walker et al. 2000). Several of these animals do not have dense hair-coats and hence ticks with short mouthparts are prone to be removed by grooming or predation by red-billed oxpeckers, *Buphagus erythrorhyncus* (Bezuidenhout & Stutterheim 1980).

Infestation of rhinos with *R. appendiculatus* nymphs and with *R. eversti eversi* adults should be regarded as incidental. *Rhipicephalus neumanni* occurs in the drier western regions of South Africa and in southern Namibia (Walker et al. 2000), and both animals that were infested were sampled in the Northern Cape province. *Rhipicephalus zumpti* is a rather rare species and is present in coastal woodland and adjacent regions in KwaZulu-Natal and the Eastern Cape province (Walker et al. 2000). It also seems to have a preference for monogastric mammals and fairly large numbers have been collected from bushpigs in north-eastern KwaZulu-Natal (Horak et al. 1991b) and now three males from two black rhinoceroses in the Eastern Cape province.

### General

Although fewer black than white rhinoceroses were sampled for ticks, they were examined in more geographic regions and hence a greater number of tick species were recovered from them. Those in Zambia alone harboured three species not collected elsewhere. The central and south eastern region of Limpopo province combined with northern Gauteng constituting the central northern provinces, was the most species-rich with seven species collected from rhinos.

Most of the ticks that were collected from rhinos in the present study are characterised by one or two features; they are ornate and/or have long or sturdy mouthparts. All the *Amblyomma* spp. collected from the rhinos are ornate and have long mouthparts. Their long mouthparts not only ensure a sturdy hold-fast on the thick hides of their hosts but also have the advantage of making them less prone to being removed by grooming or to predation by oxpeckers because of the difficulty with which they are dislodged. Their ornamentation possibly alerts oxpeckers to the fact that they will be difficult to remove. *Dermacentor rhinocerinus* is ornate and has medium length, sturdy mouthparts. The three *Hyalomma* spp. have long mouthparts and the legs of *H. glabrum* are ornate. *Rhipicephalus longiceps* has long mouthparts and *R. maculatus* is ornate and has short but sturdy mouthparts. Moreover, *R. maculatus* adults probably escape severe predation by attaching amongst the *Amblyomma* spp. on the ventral aspects of their hosts’ bodies. In addition, the colouration on the scuta of *R. maculatus* females mimics that of *A. hebraeum* females. *Rhipicephalus follis*, *R. gertrudae* and *R. simus* have short mouthparts, but with *H. truncatum*, attach to the tail and its tip within the tailbrush.

## Conclusion

The ticks that infest rhinoceroses in southern Africa are generally ornate and have long or robust mouthparts. These comprise six *Amblyomma* species and *D. rhinocerinus*, *H. glabrum* and *R. maculatus*, as well as the now possibly extinct species *Cosmiomma hippotamensis.* Amongst these ticks *A. rhinocerotis* is in danger of extinction in South Africa, while *D. rhinocerinus* is likely to survive in the north-eastern regions of the country for as long as rhinoceroses are also present. The only species that was present in all ten regions in which ticks were collected was *H. truncatum*, followed by *H. rufipes* in seven regions. The latter two species and *A. hebraeum* and *A. variegatum* are important vectors of disease to domestic livestock.
